# Methane activation by gold-doped titanium oxide cluster anions with closed-shell electronic structures†

**DOI:** 10.1039/c6sc00539j

**Published:** 2016-03-29

**Authors:** Yan-Xia Zhao, Xiao-Na Li, Zhen Yuan, Qing-Yu Liu, Qiang Shi, Sheng-Gui He

**Affiliations:** a Beijing National Laboratory for Molecular Sciences, State Key Laboratory for Structural Chemistry of Unstable and Stable Species, Institute of Chemistry, Chinese Academy of Sciences Beijing 100190 P. R. China shengguihe@iccas.ac.cn qshi@iccas.ac.cn; b University of Chinese Academy of Sciences Beijing 100049 P. R. China

## Abstract

The reactivity of closed-shell gas phase cluster anions AuTi_3_O_7_^−^ and AuTi_3_O_8_^−^ with methane under thermal collision conditions was studied by mass spectrometric experiments and quantum chemical calculations. Methane activation was observed with the formation of AuCH_3_ in both cases, while the formation of formaldehyde was also identified in the reaction system of AuTi_3_O_8_^−^. The cooperative effect of the separated Au^+^ and O^2−^ ions on the clusters induces the cleavage of the first C–H bond of methane. Further activation of the second C–H bond by a peroxide ion O_2_^2−^ leads to the formation of formaldehyde. This study shows that closed-shell species on metal oxides can be reactive enough to facilitate thermal H–CH_3_ bond cleavage and the subsequent conversion.

## Abstract 2

The complementary active sites of Au^+^ and O^2−^ ions on gold-doped titanium oxide clusters activate methane under thermal collision conditions.

## Graphical abstract

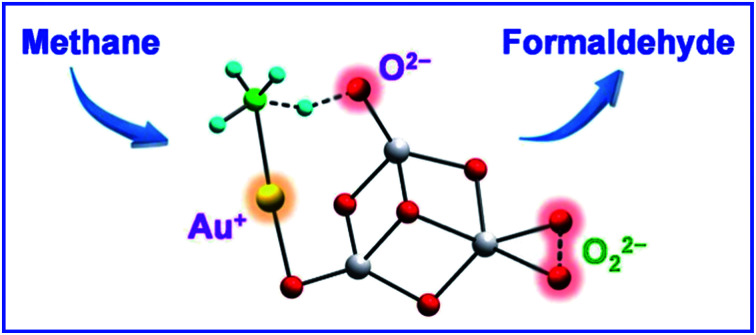

## Introduction

Methane, the major component of natural gas and shale gas, represents an important feedstock for the production of value-added chemicals.^1–6^ However, the direct conversion of methane poses a serious challenge in contemporary catalysis owing to the significant energy required for C–H bond cleavage.^1^ Many heterogeneous and homogeneous catalytic systems have been studied to transform methane, while it is challenging to uncover the elementary reactions and molecular level (ML) mechanisms associated with methane activation and conversion.^3–7^ In the last decades, model investigations of the elementary reactions between methane and gas phase atomic clusters with state-of-the-art mass spectrometric experiments and quantum chemistry calculations have been serving as an important approach to discovering the ML mechanisms of methane activation and transformation.^8–16^ The identified mechanisms can be very useful for catalyst design and optimization.^17–20^

Many atomic clusters including oxides,^8–11^ carbides,^21^ noble metals,^13–16^ and so on have been identified to be able to activate methane under thermal collision conditions. Investigations of these cluster systems have revealed three types of mechanism to activate the C–H bond of methane:

1 [MO˙^−^] + CH_4_ → [M(O–H)^−^] + CH_3_˙

2 [M] + CH_4_ → [H–M–CH_3_]

3 [ML] + CH_4_ → [M–CH_3_] + H–L

where M is usually a metal atom and L is a ligand bonded with M. The atomic oxygen radical anions (O˙^−^) were extensively identified to activate methane through hydrogen atom abstraction (Reaction (1))^8–11,22^ on many oxide clusters such as V_4_O_10_^+^,^23^ Al_8_O_12_^+^,^24^ and AuNbO_3_^+^.^25^ The coordinatively unsaturated metal atoms in naked and ligated metal species such as Au_2_^+^,^14^ Pt_*n*_^+^,^15^ PtCH_3_^+^,^26^ and TaC_*n*_^+^ (ref. 21) can activate methane through oxidative addition (Reaction (2)).^9,22,27^ The mechanism of σ-bond metathesis (Reaction (3))^9,22^ was reported for a few simple mononuclear systems such as NiF^+^ (ref. 28) and HTiO^+^ (ref. 29) in which the σ-bonded ligand can be replaced through a reaction with the σ-bond of the incoming CH_4_. Herein, we report a new mechanism of methane activation by gas phase atomic clusters (Reaction (4)):

4 [M^+^⋯O^2−^] + CH_4_ → [CH_3_–M⋯(O–H)^−^]

where the metal cation M^+^ is separated from the oxygen anion O^2−^ on polynuclear metal oxide clusters and the cooperation of the two ions with counter polarity cleaves the C–H bond of methane.

The ability of two atoms with different polarity to promote chemical activity of atomic clusters in reactions with small molecules has been previously identified in the literature. Castleman, Khanna, and their co-workers have reported that a complementary active site composed of a pair of adjacent Al atoms that respectively act as a Lewis acid and Lewis base can activate a variety of polar molecules such as water, alcohols, aldehydes, and thiols.^30–33^ Recently, we have found that a pair of non-adjacent ions Au^+^⋯O^2−^ on AuCeO_2_^+^ and AuCe_2_O_4_^+^ cations can activate the non-polar dihydrogen.^34^ However, these clusters are still not reactive enough to activate methane. This study reports that the cooperation of the separated Au^+^ and O^2−^ ions on gold-doped titanium oxide clusters AuTi_3_O_7_^−^ and AuTi_3_O_8_^−^ can bring about methane activation at thermal energies. Subsequent conversion of methane to a stable organic compound, formaldehyde, has also been identified. It is noteworthy that for the thermal activation of methane, most of the reactive clusters reported have open-shell electronic structures and the very few reactive species with closed-shell electronic structures are all mononuclear cations.^26,29^ The cluster anions were generally found to be much less reactive than the corresponding cations in the reactions with methane.^10,13,16^ For the first time, we report thermal methane activation by cluster anions with closed-shell electronic structures.

## Results

### Reactivity of AuTi_3_O_7_^−^ and AuTi_3_O_8_^−^ with methane

**Fig. 1:**
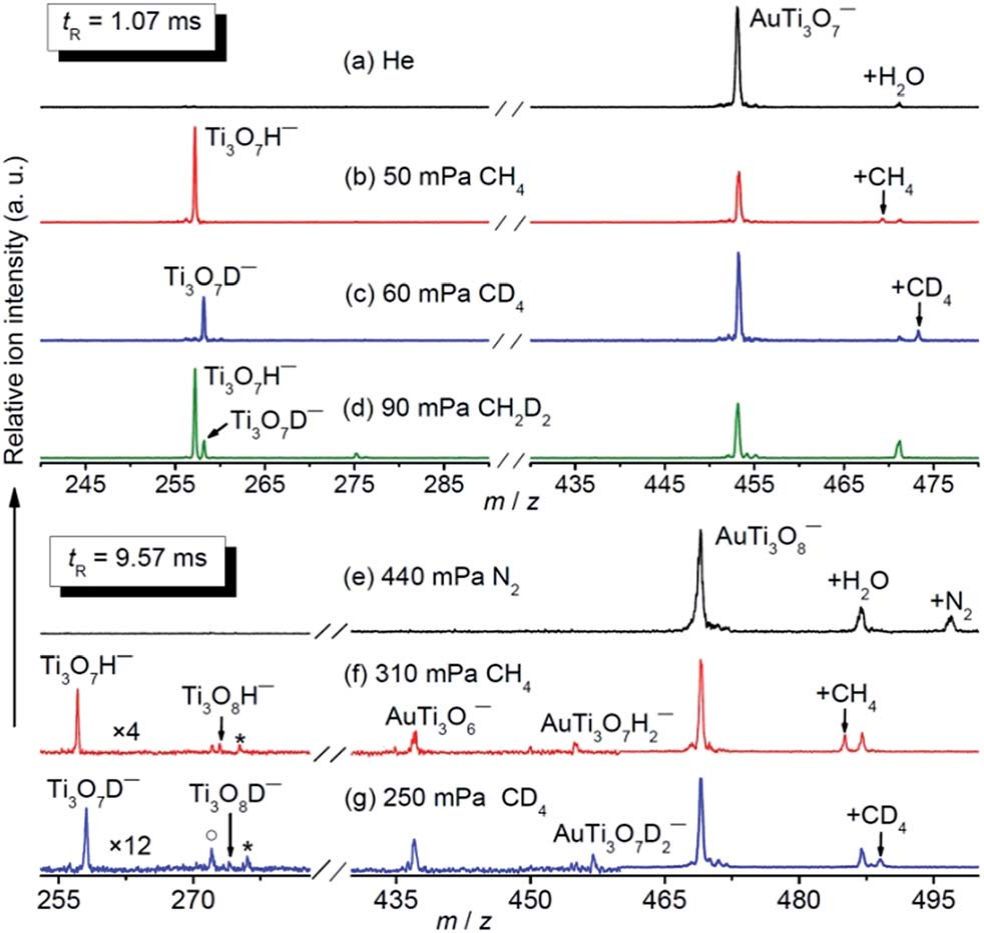
Time-of-flight mass spectra for the reactions of mass selected AuTi_3_O_7_^−^ and AuTi_3_O_8_^−^ with CH_4_ (b and f), CD_4_ (c and g), CH_2_D_2_ (d), and N_2_ (e). The peaks marked with asterisks and hollow circles in panels (f) and (g) represent water adsorption products (Ti_3_O_7_HH_2_O^−^ or Ti_3_O_7_DH_2_O^−^) and Ti_3_O_8_^−^ from collision induced dissociation, respectively. The peaks marked with +X (X = H_2_O, CH_4_, CD_4_, and N_2_) in panels (a–c) and (e–g) denote the association products with AuTi_3_O_7_^−^ and AuTi_3_O_8_^−^, respectively. The reactant gas pressures are shown. The reaction times (*t*_R_) are 1.07 ms for (a–d) and 9.57 ms for (e–g). The signal magnitudes below *m*/*z* 460 are amplified by 4 and 12 for (f) and (g), respectively.

The AuTi_3_O_7_^−^ and AuTi_3_O_8_^−^ cluster anions were prepared by a reaction of O_2_ with metal plasmas generated by laser vaporization of a solid disk compressed with Au and ^48^Ti powders. The clusters of interest were mass-selected by a quadrupole mass filter and entered into a linear ion trap reactor, where they were thermalized by collisions with a pulse of He gas (maximal instantaneous pressure around 2–4 Pa) and then interacted with a pulse of CH_4_, CD_4_, or CH_2_D_2_ for a period of time.^35^ Upon the interaction of AuTi_3_O_7_^−^ with 50 mPa CH_4_ for about 1.07 ms (Fig. 1b), a strong product peak that can be assigned to Ti_3_O_7_H^−^ was observed, suggesting the following reaction channel:

5 AuTi_3_O_7_^−^ + CH_4_ → Ti_3_O_7_H^−^ + AuCH_3_

The isotopic labelling experiments with CD_4_ (Fig. 1c) and CH_2_D_2_ (Fig. 1d) confirmed the above reaction. The generation of Ti_3_O_7_D^−^ was observed from AuTi_3_O_7_^−^ + CD_4_ while both Ti_3_O_7_H^−^ and Ti_3_O_7_D^−^ were produced from AuTi_3_O_7_^−^ + CH_2_D_2_. The inter- and intra-molecule isotopic effects were apparently observed. In addition to Reaction (5), a minor association reaction channel generating AuTi_3_O_7_CH_4_^−^ (4% of the total product ions) was also observed.

The AuTi_3_O_8_^−^ cluster is much less reactive than AuTi_3_O_7_^−^ and a longer reaction time and higher methane pressures were used for AuTi_3_O_8_^−^ + CH_4_ (Fig. 1f), in which the generation of Ti_3_O_7_H^−^, Ti_3_O_8_H^−^, AuTi_3_O_6_^−^, and AuTi_3_O_7_H_2_^−^ was observed and the assignments were confirmed by the experiments with CD_4_ and CH_2_D_2_ (Fig. 1g and S1 ESI†). In a reference experiment with N_2_ (Fig. 1e), the AuTi_3_O_6_^−^ product was not generated, indicating that this product cluster in Fig. 1f and g was due to a chemical reaction rather than collision induced dissociation (CID, such as AuTi_3_O_8_^−^ + CH_4_ → AuTi_3_O_6_^−^ + O_2_ + CH_4_). In addition to molecular association, the following four reaction channels are suggested by the experiments:

6 AuTi_3_O_8_^−^ + CH_4_ → Ti_3_O_8_H^−^ + AuCH_3_

7 AuTi_3_O_8_^−^ + CH_4_ → AuTi_3_O_7_H_2_^−^ + CH_2_O

8 AuTi_3_O_8_^−^ + CH_4_ → Ti_3_O_7_H^−^ + AuCH_3_O (CH_2_O + AuH)

9 AuTi_3_O_8_^−^ + CH_4_ → AuTi_3_O_6_^−^ + CH_4_O_2_ (CH_2_O + H_2_O)

The branching ratios of generating AuTi_3_O_8_CH_4_^−^ (41%), Ti_3_O_7_H^−^ (35%), and AuTi_3_O_6_^−^ (15%) are much larger than those of AuTi_3_O_7_H_2_^−^ (6%) and Ti_3_O_8_H^−^ (3%) (Fig. 1f).

An atomic cluster often has different structural isomers with very different reactivities.^29^ The analysis of the methane-pressure dependent reactivity indicated that (87 ± 1)% of the experimentally generated AuTi_3_O_7_^−^ ions (Fig. S2†) and only (37 ± 4)% of the AuTi_3_O_8_^−^ ions (Fig. S3†) were reactive with CH_4_. For the reactive component of AuTi_3_O_7_^−^, the pseudo first-order rate constant (*k*_1_) of Reaction (5) is (7.7 ± 2.3) × 10^−11^ cm^3^ per molecule per second, corresponding to a reaction efficiency (*Φ*)^36^ of (7.7 ± 2.3)%. The intra and inter-molecular kinetic isotope effects (KIEs) amount to 5.0 ± 1.1 and 4.5 ± 1.3, respectively. For the reactive component of AuTi_3_O_8_^−^, the summed *k*_1_ value of Reactions (6)–(9) is (1.0 ± 0.3) × 10^−12^ cm^3^ per molecule per second [*Φ* = (0.1 ± 0.03)%]. The inter-molecular KIE amounts to 4.0 ± 1.3.

### Reaction mechanisms of AuTi_3_O_7_^−^ and AuTi_3_O_8_^−^ with CH_4_

Density functional theory (DFT) calculations at the TPSS level, which shows the overall best performance in calculating several critical bond energies among the 18 tested methods (Table S1, ESI†), have been conducted to explore the detailed reaction mechanisms. A Fortran code based on the genetic algorithm was used to search the global minimum structures of AuTi_3_O_7_^−^ as well as AuTi_3_O_8_^−^ clusters with different spin multiplicities.^37^ To determine the lowest-energy isomers of AuTi_3_O_7_^−^ and AuTi_3_O_8_^−^, further single-point energy calculations with a high-level quantum chemistry method of a restricted coupled-cluster method with single, double, and perturbative triple excitations [RCCSD(T)] have also been performed for the TPSS optimized structures. In the reaction pathway of AuTi_3_O_8_^−^ + CH_4_, concomitant cleavage and formation of several chemical bonds are involved in important transition states. It is known that commonly used density functionals do not correctly describe the long- and mid-range dispersion interactions, which can influence the chemical reaction energies.^38^ Thus, TPSS functional calculated energies with dispersion corrections^38^ are given throughout the reaction pathways.

**Fig. 2:**
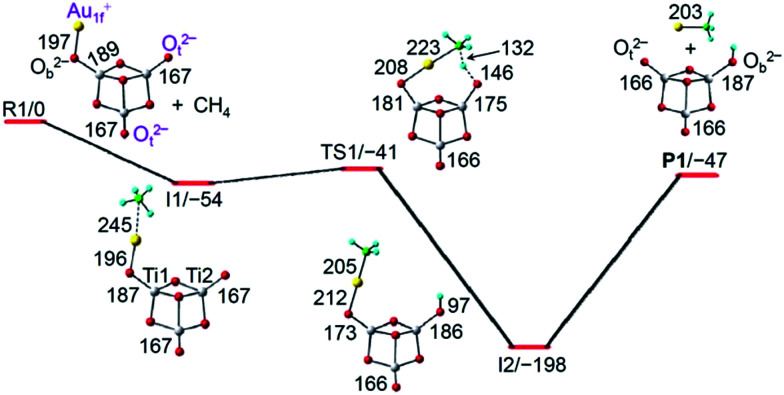
TPSS functional calculated potential energy profile for AuTi_3_O_7_^−^ + CH_4_ → Ti_3_O_7_H^−^ + AuCH_3_. All of the species are in a singlet spin state. The dispersion corrected energies of the reaction intermediates (I1 and I2), transition state (TS1), and products (P1) with respect to the separated reactants (R1) are given in kJ mol^−1^. Bond lengths are given in pm.

The lowest-lying isomer of AuTi_3_O_7_^−^ (Fig. 2 and S5†) has two terminally-bonded oxygen anions (O_t_^2−^, −0.62e) and a one-fold coordinated gold cation (Au_1f_^+^, +0.42e). It is noteworthy that the superscripts “2−” of O^2−^ and “+” of Au^+^ denote the formal oxidation states rather than the net charges on the atoms. As marked in Fig. 2, the Au_1f_^+^ cation is separated from the O_t_^2−^ anions. The Au-side of AuTi_3_O_7_^−^ is the least negatively charged, so it is expected that the Au_1f_^+^ atom traps CH_4_ to form the encounter complex I1 with a significant binding energy (54 kJ mol^−1^). Then the CH_4_ is delivered to be close to a O_t_^2−^ ion (TS1) so that the cleavage of one C–H bond and the concomitant formation of one Au–CH_3_ bond and one O–H bond can take place. Such a process (I1 → TS1 → I2) is subject to an energy barrier of 13 kJ mol^−1^ which is surmountable by the binding energy of I1. The formation of I2 can release a high energy of 198 kJ mol^−1^, which is enough to evaporate the AuCH_3_ species rather than Au + CH_3_ (endothermic by 244 kJ mol^−1^) from the reaction complex to form the experimentally observed Ti_3_O_7_H^−^ cluster (Fig. 1b).

The gold atom in the lowest-lying isomer of AuTi_3_O_7_^−^ is one-fold coordinated (Fig. 2). In a low-lying isomer of AuTi_3_O_7_^−^ (Fig. S5†), the Au^+^ ion can be two-fold coordinated (Au_2f_^+^). Such a cluster anion with Au_2f_^+^ can hardly trap (binding energy is only 8 kJ mol^−1^) the reactant molecule CH_4_. The activation of CH_4_ by AuTi_3_O_7_^−^ with the Au_2f_^+^ is subject to an additional transformation so that the Au_2f_^+^ ion becomes Au_1f_^+^ (as in I1 of Fig. 2). The Au_2f_^+^ → Au_1f_^+^ transformation has an overall positive barrier (3 kJ mol^−1^) which hinders subsequent methane activation, suggesting that the AuTi_3_O_7_^−^ isomer with Au_2f_^+^ corresponds to the un-reactive component (13%) of AuTi_3_O_7_^−^ in the experiments.

**Fig. 3:**
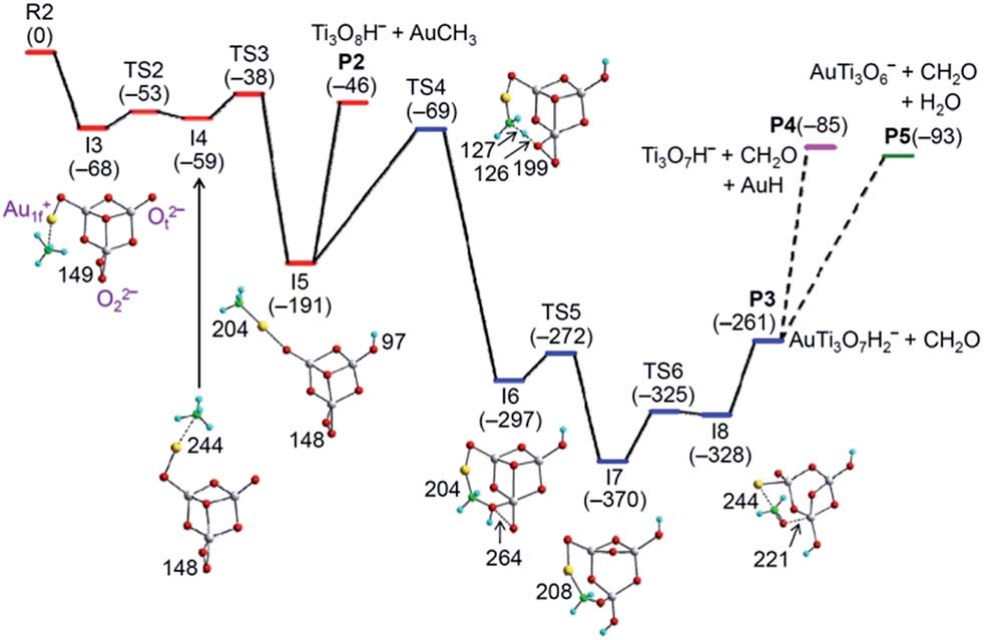
TPSS functional calculated potential energy profile for AuTi_3_O_8_^−^ + CH_4_ to generate the products Ti_3_O_8_H^−^ + AuCH_3_ (P2), AuTi_3_O_7_H_2_^−^ + CH_2_O (P3), Ti_3_O_7_H^−^ + CH_2_O + AuH (P4), and AuTi_3_O_6_^−^ + CH_2_O + H_2_O (P5). All of the species are in a singlet spin state. The dispersion corrected energies of the reaction intermediates (I3–I8), transition states (TS2–TS6), and products (P2–P5) with respect to the separated reactants (R2) are given in kJ mol^−1^. Bond lengths are given in pm.

When one of the O_t_^2−^ ions in the lowest-lying isomer of AuTi_3_O_7_^−^ is replaced by a peroxide unit (O_2_^2−^), a low-lying isomer of AuTi_3_O_8_^−^ with Au_1f_^+^ and O_t_^2−^ ions can be formed (see I3 of Fig. 3). However, this isomer with Au_1f_^+^ is less stable by 24 kJ mol^−1^ than the lowest-lying isomer of AuTi_3_O_8_^−^ that contains an Au_2f_^+^ ion (Fig. S6†). The activation of CH_4_ by the lowest-lying isomer of AuTi_3_O_8_^−^ also involves the Au_2f_^+^ → Au_1f_^+^ conversion which is hindered by an overall positive reaction barrier (1 kJ mol^−1^). As a result, this lowest-lying isomer with Au_2f_^+^ accounts for the 63% un-reactive component of the experimentally generated AuTi_3_O_8_^−^ ions. The 37% reactive ions can then be assigned to the low-lying isomer with Au_1f_^+^ and the reaction mechanism is shown in Fig. 3.

Similarly to AuTi_3_O_7_^−^ + CH_4_ in Fig. 2, the Au_1f_^+^ on AuTi_3_O_8_^−^ traps CH_4_ and delivers CH_4_ to be close to the O_t_^2−^ ion for C–H bond cleavage (I4 → TS3 → I5, Fig. 3). The large amount of exothermic energy released (191 kJ mol^−1^) can evaporate the AuCH_3_ unit to produce Ti_3_O_8_H^−^ (I5 → P2, Reaction (6)). Alternatively, I5 can overcome the energy barrier (122 kJ mol^−1^, I5 → TS4) involving the activation of a second C–H bond by the O_2_^2−^ unit (I5 → TS4 → I6) to produce a neutral CH_2_O molecule and AuTi_3_O_7_H_2_^−^ ions (I6 → TS5 → I7 → TS6 → I8 → P3, Reaction (7)). The formation of AuTi_3_O_7_H_2_^−^ and CH_2_O is highly exothermic (Δ*H*_0_ = −261 kJ mol^−1^), so the resulting AuTi_3_O_7_H_2_^−^ has enough internal energy to evaporate AuH and H_2_O (Fig. S7†) to form the product ions Ti_3_O_7_H^−^ (Reaction (8)) and AuTi_3_O_6_^−^ (Reaction (9)), respectively. It can be seen that Reactions (7)–(9) all involve the generation of formaldehyde (CH_2_O) and the DFT calculations correctly predict that Reactions (6)–(9) are all kinetically and thermodynamically favorable. Furthermore, the lower energy of TS4 (−69 kJ mol^−1^) than that of P2 (−46 kJ mol^−1^) can well rationalize the experimental branching ratios that show Reaction (6) as a minor channel.

In the reaction of AuTi_3_O_8_^−^ with CH_4_, the Au_1f_^+^ cation can also deliver CH_4_ to be close to the O_2_^2−^ anion to activate the first C–H bond of methane (Fig. S9†). Subsequent transformation to form the intermediate I5 is kinetically less favorable than the reaction path of Fig. 3. A reaction path to form I7 (Fig. S10†) is slightly more favorable than the path of Fig. 3 kinetically (−45 kJ mol^−1^*versus* −38 kJ mol^−1^ for the critical transition states). This alternative path has a very deep potential well (209 kJ mol^−1^) which can hinder the further transformation of the reaction complex into separate products and leads to the formation of the association species AuTi_3_O_8_CH_4_^−^. This result is consistent with the experimental observation that molecular association is a major reaction channel (41%) for AuTi_3_O_8_^−^ + CH_4_ (Fig. 1f).

## Discussion

Many metal oxide clusters including homo-nuclear (M_*x*_O_*y*_^±^) and hetero-nuclear oxide clusters (M^1^_*x*1_M^2^_*x*2_O_*y*_^±^) have been found to react with methane under thermal collision conditions.^8–11,22–25^ All of the reactive oxide clusters were open-shell systems with oxygen radical centers and methane activation primarily followed Reaction (1). Recently, it has been demonstrated that the PtAl_2_O_4_^−^ cluster can activate CH_4_ through Reaction (2) and then the oxygen radical accepts the transferred H atom.^39^ Herein, the AuTi_3_O_7_^−^ and AuTi_3_O_8_^−^ clusters are closed-shell systems (without radical oxygen species) and they activate methane through the new mechanism, Reaction (4). Previously, a few positively charged mononuclear species^26,29^ with closed-shell electronic structures were shown to activate methane through Reactions (2) and (3).

The observed reactivity of AuTi_3_O_7_^−^ and AuTi_3_O_8_^−^ with methane (Reactions (5)–(9)) can be closely related to the extraordinary properties of gold, and results from the strong relativistic effect on this element.^40^ The high electro-negativity of gold leads to a rather weak Au–O chemical bond (bond energy of 219 kJ mol^−1^)^41^ so that the O–Au_1f_^+^ bond can be flexible for the delivery of CH_4_ to be close to O^2−^ (Fig. 2 and 3) or O_2_^2−^ (Fig. S9†) for C–H activation. Moreover, the analogous Au/H^42^ results in relatively strong Au–CH_3_ (232 kJ mol^−1^ by the TPSS functional) and Au–H (292 kJ mol^−1^ by the TPSS functional) bonds so that the evaporation of AuCH_3_ (Reactions (5) and (6)) and AuH (Reaction (8)) from the reaction complex is possible. Upon generation of the CH_2_O moiety in AuTi_3_O_8_^−^ + CH_4_, the Au atom is bonded with the metal atom Ti (I8 of Fig. 3) and the gold atom becomes negatively charged (−0.20e), which is also a result of the relativistic effect.^40,43,44^

In addition to the extraordinary properties of gold, the co-participation of both Au_1f_^+^ and O_t_^2−^ ions is very important for the activation of methane (Fig. 2 and 3). The reaction paths for the C–H bond cleavage of CH_4_ by the single Au_1f_^+^ cation and a single O_t_^2−^ anion of AuTi_3_O_7_^−^ have been also followed. These processes are subject to very high energy barriers (Fig. S11†). In addition, the reaction of AuTi_3_O_7_^−^ with CH_4_ on the triplet potential energy surface has also been calculated. It turned out that all of the triplet reactants, intermediates, and transition states are much higher (>100 kJ mol^−1^) in energy than the corresponding singlet counterparts. As a result, the cooperative activation by the Au_1f_^+^ and O_t_^2−^ ions, as shown in Fig. 2, is the only mechanism of methane activation by AuTi_3_O_7_^−^.

To explore the excellent ability of the cooperative Au_1f_^+^ and O_t_^2−^ ions to promote methane activation by the closed-shell cluster anions of AuTi_3_O_7_^−^ and AuTi_3_O_8_^−^ under thermal collision conditions, variation of the geometrical structures of the reaction intermediates and the change of natural charge (Table S2†) as well as the Wiberg bond order (Table S3†) of critical atoms and chemical bonds have been analyzed for the reaction system of AuTi_3_O_7_^−^ + CH_4_ (Fig. 2). During the course of C–H bond cleavage (I1 → TS1 → I2), the electron population on the CH_3_ group and Au atom increases (−0.11e → −0.41e for CH_3_ and +0.44e → +0.28e for Au) and that on the transferring H decreases (+0.18e → +0.47e), indicating that the C–H bond may be cleaved in a heterolytic manner and the reaction may follow a Lewis acid–base pair mechanism taking into account that the separated Au_1f_^+^ and O_t_^2−^ ions in AuTi_3_O_7_^−^ can be considered as a Lewis acid and Lewis base, respectively. However, the change in the natural charges of the CH_3_ moiety and H is not very large (around 0.3e), additionally, the charge increase on the H is normal for a bond conversion of C–H → O–H. In contrast to the Lewis acid–base mechanism, another mechanism of the flexible switch of the roles of the two O_t_ atoms in [Ti_3_O_7_]^2−^ which enables the favorable C–H activation is proposed from a bonding point of view. The reactant of AuTi_3_O_7_^−^ can be viewed as Au^+^[Ti_3_O_7_]^2−^, in which Au^+^ (+0.42e) is attached to one of the O_t_ in [Ti_3_O_7_]^2−^ (−1.42e) with a bond strength of 1058 kJ mol^−1^ and the O_t_ becomes a bridging-bonded oxygen (O_b_, as marked in Fig. 2). During the C–H activation, such O_b_ releases gold to bond with the CH_3_ moiety and the O_b_ itself switches to O_t_ after AuCH_3_ evaporation (Fig. 2). At the same time, a different O_t_ in [Ti_3_O_7_]^2−^ switches to O_b_ after attaching to H^+^ (+0.46e) with a much stronger bond strength of 1710 kJ mol^−1^ (Fig. 2). Thus, the overall increased bond strengths in the products drive the C–H activation thermodynamically. The switch of the roles of the two O_t_ in [Ti_3_O_7_]^2−^ can also be evidenced by the change of the Wiberg bond order of Ti–O_t_ (Table S3†). The Ti1–O_t_ and Ti2–O_t_ bonds (Fig. 2) gradually switch from single/double bonds (187/167 pm) in the reactant to double/single bonds (166/187 pm) in the product. This mechanism suggests that the C–H bond may be cleaved in a homolytic manner, namely, hydrogen atom transfer (HAT).

The identification of the Lewis acid–base mechanism or HAT for AuTi_3_O_7_^−^ + CH_4_ relies on the transfer mode of one electron (e^−^) and one proton (H^+^) of a H atom. HAT is characterized by the transfer of the electron and proton to a single site. In contrast, a Lewis acid–base mechanism corresponds to the transfer of the electron and proton to different acceptor sites. Such a transfer mode can be called an electron–proton transfer (EPT).^45^ It was proposed that a key element in the theoretical characterization of the mechanisms of proton and electron transfer is the formulation of their localized diabatic states.^46^ However, the electron and proton described by standard quantum mechanical methods tend to be delocalized, and the analysis of the electron or proton acceptors such as a molecular orbital or a chemical bond depends on the adopted computational level of theory.^45^ Consequently, it is hard to distinguish exactly the two proposed mechanisms of AuTi_3_O_7_^−^ + CH_4_ at the present level of theory. More advanced methods such as multistate DFT, in which the electron and proton localized diabatic configurations can be constructed through block-localization of Kohn–Sham orbitals, should be employed to study the potential energy surfaces of the HAT and EPT, which may provide clues to recognize the mechanistic details of our reaction systems.

## Conclusions

The reactions of methane with negatively charged titanium oxide clusters doped with single gold atoms, AuTi_3_O_7_^−^ and AuTi_3_O_8_^−^, have been identified by mass spectrometry and quantum chemistry calculations. To the best of our knowledge, this is the first example of the thermal activation and transformation of methane by atomic cluster anions with closed-shell electronic structures. Unlike the previously reported three general mechanisms including hydrogen atom abstraction, oxidative addition, and σ-bond metathesis for methane activation by atomic clusters, the cooperation of the separated Au^+^ and O^2−^ induces the cleavage of the first C–H bond of methane for AuTi_3_O_7_^−^ and AuTi_3_O_8_^−^ clusters. Further activation of the second C–H bond of methane on the oxygen-rich system of AuTi_3_O_8_^−^ leads to the formation of the stable organic compound formaldehyde (CH_2_O). The observed unique reactivity of AuTi_3_O_7_^−^ and AuTi_3_O_8_^−^ toward methane results from the strong relativistic effect on the gold element. This study not only serves as an important step in understanding that closed-shell species on metal oxides can be reactive enough to facilitate thermal H–CH_3_ bond cleavage, but also provides molecular-level insights into the design of active sites on metal oxide supported gold catalysts to activate and transform methane.

## Supplementary Material

SC-007-C6SC00539J-s001
