# Supplementary material for: Methane activation by gold-doped titanium oxide cluster anions with closed-shell electronic structures
Source: Chem Sci. 2016 Mar 29;7(7):4730–5. doi: 10.1039/c6sc00539j (PMC6016522; doi:10.1039/c6sc00539j)
Supplement: SC-007-C6SC00539J-s001 [file SC-007-C6SC00539J-s001.pdf]

Electronic Supplementary Material (ESI) for Chemical Science.
This journal is © The Royal Society of Chemistry 2016

Supplementary Information for:

Methane Activation by Gold-Doped Titanium Oxide Cluster Anions with Closed-Shell Electronic Structures

Yan-Xia Zhao[1], Xiao-Na Li[1], Zhen Yuan[1,2], Qing-Yu Liu[1,2], Qiang Shi[1,*], and Sheng-Gui He[1,*]

[1]Beijing National Laboratory for Molecular Sciences, State Key Laboratory for Structural Chemistry of Unstable and Stable Species, Institute of Chemistry, Chinese Academy of Sciences, Beijing 100190, P. R. China.

[2]University of Chinese Academy of Sciences, Beijing 100049, P. R. China

Table of Contents

1. Experimental and computational methods (pages S2 and S3).

2. Additional experimental results.

2.1. Cluster reactivity of $AuTi_3O_8^-$ (page S4).

2.2. Reaction kinetics for $AuTi_3O_7^-$ + CH_4 (pages S5 and S6).

2.3. Reaction kinetics for $AuTi_3O_8^-$ + CH_4 (page S7).

2.4. An experiment to verify that the reactant cluster ions are thermalized (page S8).

3. Additional computational results.

3.1. Isomeric structures of clusters (pages S9-S10).

3.2. Additional reaction pathways for the reactions of $AuTi_3O_8^-$ + CH_4 and $AuTi_3O_7^-$ + CH_4 (pages S11-S15).

3.3. Natural bond orbital analysis of the charge and wiberg bond order of critical atoms and chemical bonds involved in the species of Figure 2 in the main text (page S16).

3.4 Coordinates for the low-lying energy isomers of $AuTi_3O_7^-$ and $AuTi_3O_8^-$ clusters shown in Figs. S5 and S6 (pages S17-S23).

1. Experimental and computational methods.

The $Au^{48}Ti_xO_y^-$ cluster anions were generated by the reaction of O_2 (1% O_2 seeded in He carrier gas with a backing pressure of 5 standard atmospheres) with metal plasmas generated by laser (energy of 5−8 mJ per pulse at 532 nm and frequency of 10 Hz) vaporization of a solid metal disk compressed with isotope-enriched Ti powder (99.32% ^{48}Ti, Isoflex, San Francisco) and Au powder (Au/Ti mole ratio = 1/1). The clusters of interest ($Au^{48}Ti_3O_7^-$ and $Au^{48}Ti_3O_8^-$) were mass-selected by a quadrupole mass filter and entered into a linear ion trap reactor, where they were thermalized by collisions with a 5−8 Pa He gas for about 1.0 ms and then interacted with a pulse of CH_4, CD_4, or CH_2D_2 for a period of time. A reflectron time-of-flight mass spectrometer was used to detect the cluster ions.[1]

The density functional theory (DFT) calculations using Gaussian 09 program[2] were carried out to investigate the structures of $AuTi_3O_7^-$ and $AuTi_3O_8^-$ clusters and the reaction mechanisms with CH_4. The bond dissociation energies of AuO, AuH, AuC^+, TiO, H−CH_3, OH, O_2 and CO were computed by various functionals and compared with available experimental data (Table S1). With the TZVP[3] basis sets for Ti, O, C, and H atoms and a D95V basis set[4] combined with the Stuttgart/Dresden relativistic effective core potential (denoted as SDD in Gaussian software) for Au atom, the TPSS functional[5] is the overall best (see Table S1.) and even better than the popular hybrid B3LYP functional[6-8] which was previously used to study the Au−Ti−O cluster structures,[9] so the results by TPSS are given throughout the main text. A Fortran code based on the genetic algorithm[10] was used to search the global minimum structures of $AuTi_3O_7^-$ and $AuTi_3O_8^-$ clusters with different spin multiplicities. To determine reliable relative energies of low-lying isomeric structures, the single-point energy calculations at the high-level of restricted coupled-cluster method with single, double, and perturbative triple excitations method [RCCSD(T)][11-13] were performed at the TPSS optimized geometries. The reaction mechanism calculations involved geometry optimization of reaction intermediates and transition states. The transition states were optimized with the Berny algorithm.[14] Intrinsic reaction coordinates calculations were performed to check that a transition state connects two appropriate local minima. The zero-point vibration corrected energies (ΔH_0) were reported. The natural bond orbital (NBO) analysis was performed with NBO 3.1.[15]

Table S1. DFT calculated and experimental bond dissociation energies of AuO, AuH, AuC^+, TiO, H–CH_3, OH, O_2 and CO. The values are in unit of kJ mol^{-1}.

		Au–O	Au–H	Au^+–C	Ti–O	H–CH_3	O–H	O–O	C–O
Experiments	Value	219	314	324	663	438	428	493	1076
	Reference	16	17	18	19	20	20	21	22
Pure Functionals	**TPSS**	**216**	**297**	**338**	**717**	**432**	**421**	**510**	**1040**
	BLYP	226	289	352	727	429	4321	548	1073
	BP86	236	304	379	759	440	446	573	1097
	BPW91	222	286	358	741	424	427	557	1079
	M06L	196	275	291	676	431	408	499	1054
	PBE	238	291	374	763	430	433	580	1102
	BPBE	223	286	360	741	424	426	559	1080
Hybrid Functionals	B3LYP	179	278	279	643	432	425	497	1042
	B1LYP	163	270	254	727	426	415	466	1019
	X3LYP	178	278	276	636	432	424	491	1041
	B1B95	170	273	268	636	437	415	497	1052
	B3P86	190	291	300	683	443	438	519	1067
	B3PW91	176	276	282	666	428	419	499	1044
	PBE1PBE	172	271	271	645	425	414	495	1043
	M05	161	258	204	621	433	415	486	1051
	M06	163	264	244	623	434	415	478	1056
	M052X	161	276	229	537	434	416	473	1047
	M062X	140	249	214	565	434	419	482	1058

2. Additional experimental results.

2.1. Cluster reactivity of $AuTi_3O_8^-$.

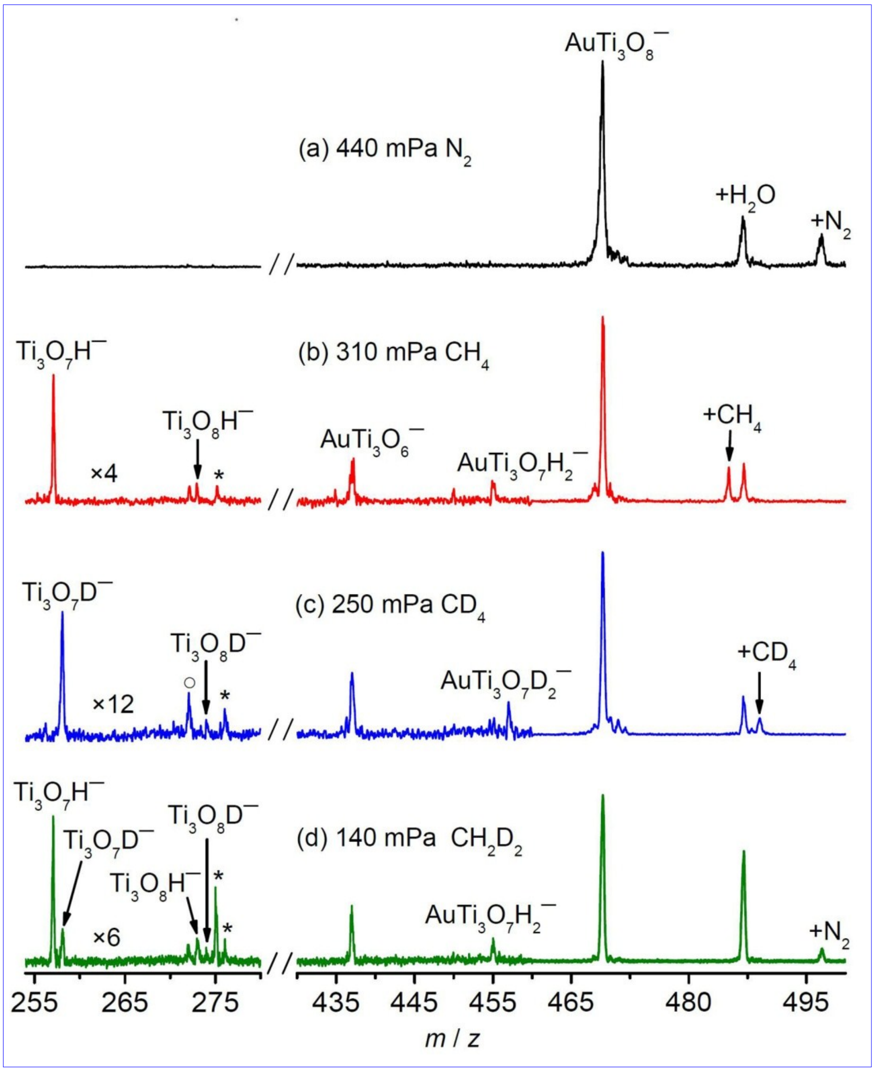

Fig. S1. TOF mass spectra for the reactions of mass selected $AuTi_3O_8^-$ with N_2 (a), CH_4 (b), CD_4 (c), and CH_2D_2 (d). The peaks marked with asterisk and hollow circle in panels (b) and (d) represent water adsorption products ($Ti_3O_7HH_2O^-$ or $Ti_3O_7DH_2O^-$) and $Ti_3O_8^-$ from collision induced dissociation, respectively. The peaks marked with +X (X = H_2O, CH_4, CD_4, and N_2) in panels (a-d) denote adsorption products by $AuTi_3O_8^-$. The reactant gas pressures are shown. The reaction time is 9.57 ms for (a-d). The signal magnitudes below *m*/*z* 460 are amplified by 4, 12, and 6 for (b), (c), and (d), respectively.

2.2. Reaction kinetics for $AuTi_3O_7^-$ + CH_4.

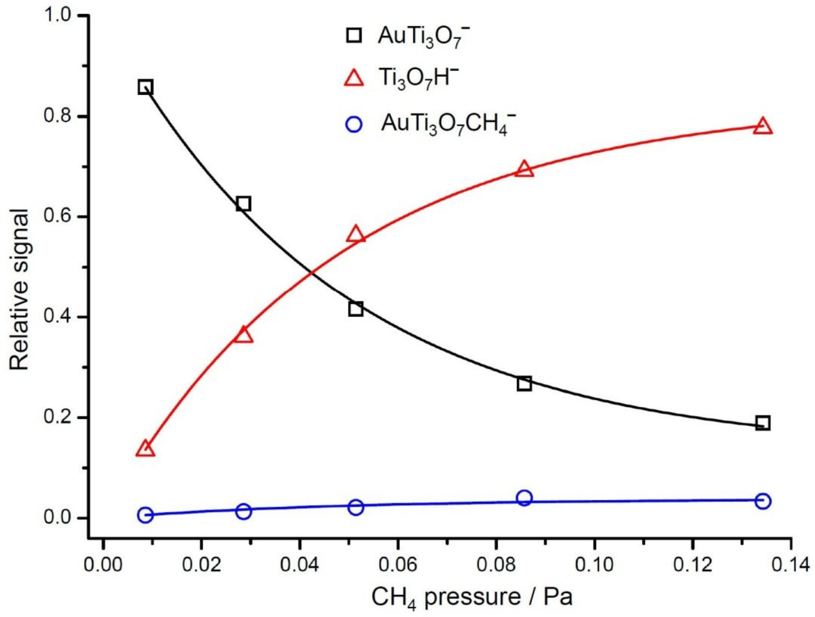

Fig. S2. Variation of ion intensities with respect to the CH_4 pressures in the reaction of $AuTi_3O_7^-$ with CH_4. The solid lines are fitted to the experimental data points by using the equations derived with the approximation of the pseudo-first-order reaction mechanism for the reactive species (Equation 11).

In reaction of $AuTi_3O_7^-$ + CH_4, both $AuCH_3$ evaporation channel (Reaction 5 in main text) and methane association channel (Reaction 10) were observed:

$$AuTi_3O_7^- + CH_4 \rightarrow Ti_3O_7H^- + AuCH_3 \quad (5)$$

$$AuTi_3O_7^- + CH_4 \rightarrow AuTi_3O_7CH_4^- \quad (10)$$

Fig. S2 plots the signal variation of the reactant and product ions with respect to the CH_4 pressure (P). The relative intensity of the reactant cluster $AuTi_3O_7^-$ (I_R) deceases from about 1.0 to 0.2 when the P value increases from 0 to about 0.13 Pa. The decrease of I_R gradually becomes subdued with the increase of P, indicating that some of the experimentally generated $AuTi_3O_7^-$ ions were inert toward CH_4. It turned out that the I_R value could be well fitted by the following Equation:

$$I_R = x_{\text{inert}} + (1 - x_{\text{inert}}) \times \exp(-k_1 \frac{P}{k_B T} t_R) \quad (11)$$

in which x_{inert} is the relative intensity of the un-reactive component of $AuTi_3O_7^-$ and k_1 is the pseudo-first-order rate constant of the reactive component of $AuTi_3O_7^-$, k_B is the Boltzmann constant, T is the temperature (≈ 300 K), and t_R is the reaction time (≈ 1.07 ms).

The x_{inert} and k_1 in Equation 11 were determined to be (13 ± 1) % and (8.04 ± 0.22) × 10^{-11} cm^3 $molecule^{-1}$ s^{-1}, respectively. The uncertainties ± 1% and ± 0.22 × 10^{-11} cm^3 $molecule^{-1}$ s^{-1} are one-standard errors in the least-square fitting. It can be seen that (87 ± 1) % of the experimentally generated $AuTi_3O_7^-$ ions were reactive with CH_4 and their relative intensity follows a single exponential decay. It is noteworthy that the absolute $k_1(AuTi_3O_7^- + CH_4)$ value (8.04 × 10^{-11} cm^3 $molecule^{-1}$ s^{-1}) can be over- or under-estimated by 30% due to systematical errors in determining the reaction time t_R and pressure P. The branching ratio of Reaction (5) accounts for 96% and that of Reaction (10) accounts for 4%.

2.3. Reaction kinetics for $AuTi_3O_8^-$ + CH_4.

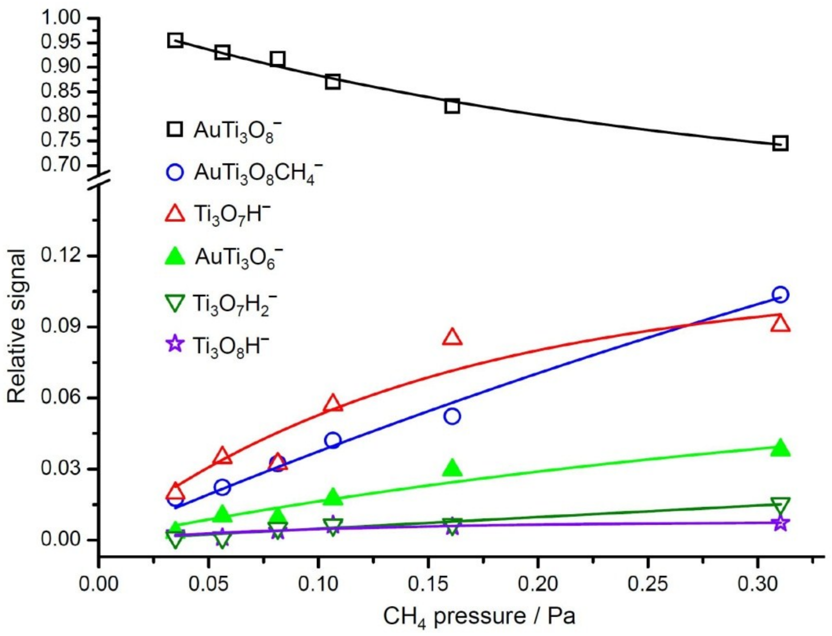

Fig. S3. Variation of ion intensities with respect to the CH_4 pressures in the reaction of $AuTi_3O_8^-$ with CH_4. The solid lines are fitted to the experimental data points by using the equations derived with the approximation of the pseudo-first-order reaction mechanism for the reactive species (Equation 11).

Five reaction channels are observed in the reaction of $AuTi_3O_8^-$ + CH_4:

$AuTi_3O_8^- + CH_4 \rightarrow Ti_3O_8H^- + AuCH_3$ (6)

$AuTi_3O_8^- + CH_4 \rightarrow AuTi_3O_7H_2^- + CH_2O$ (7)

$AuTi_3O_8^- + CH_4 \rightarrow Ti_3O_7H^- + AuOCH_3$ (CH_2O + AuH) (8)

$AuTi_3O_8^- + CH_4 \rightarrow AuTi_3O_6^- + CH_4O_2$ (CH_2O + H_2O) (9)

$AuTi_3O_8^- + CH_4 \rightarrow AuTi_3O_8CH_4^-$ (12)

Fig. S3 plots the signal variation of the reactant and product ions with respect to the CH_4 pressure (P). The decrease of I_R (1.0 → 0.8) also gradually becomes subdued with the increase of P (0 → 0.3), indicating that the un-reactive component of $AuTi_3O_8^-$ ions was also experimentally generated. According to Equation 11, the values of x_{inert} and k_1($AuTi_3O_8^-$ + CH_4) were determined to be (63 ± 4) % and (1.66 ± 0.25) × 10^{-12} cm^3 $molecule^{-1}$ s^{-1}, respectively. It can be seen that only (37 ± 4) % of the experimentally generated $AuTi_3O_8^-$ ions were reactive with CH_4. The total branching ratio of Reactions (6)-(9) accounts for 63% and that of Reaction (12) accounts for 37%.

2.4. An experiment to verify that the reactant cluster ions are thermalized.

To verify that the reactant cluster ions are thermalized before the reaction through the collisions with He atoms, an comparison experiment has been conducted for reaction of $AuTi_3O_7^-$ with CH_4 under the condition of four different cooling times: 0.85 ms, 1.15 ms, 1.45 ms, and 1.85 ms as shown in Fig. S4 (note that the He gas pulse is longer than 3 ms in our experiment). Other conditions such as the cluster generation parameters, reaction time (1.07 ms), and CH_4 pressure (95 mPa) are identical. The relative intensity of $AuTi_3O_7^-$ before (I_0) and after (I_R) reactions are analyzed. It turns out that the $\ln(I_0/I_R)$ values (from Fig. S4) under four different cooling times are identical (1.47±0.05) within the experimental uncertainties. Thus, it is certain that the reactant cluster ions are thermalized even with cooling time of only 0.85 ms. To keep the relative intensities of the minor products such as $AuTi_3O_7H_2O^-$ (Fig. S4) from residual H_2O as low as possible, the data shown in Figs. 1 and S1 are collected under the condition of short cooling time (0.85 ms).

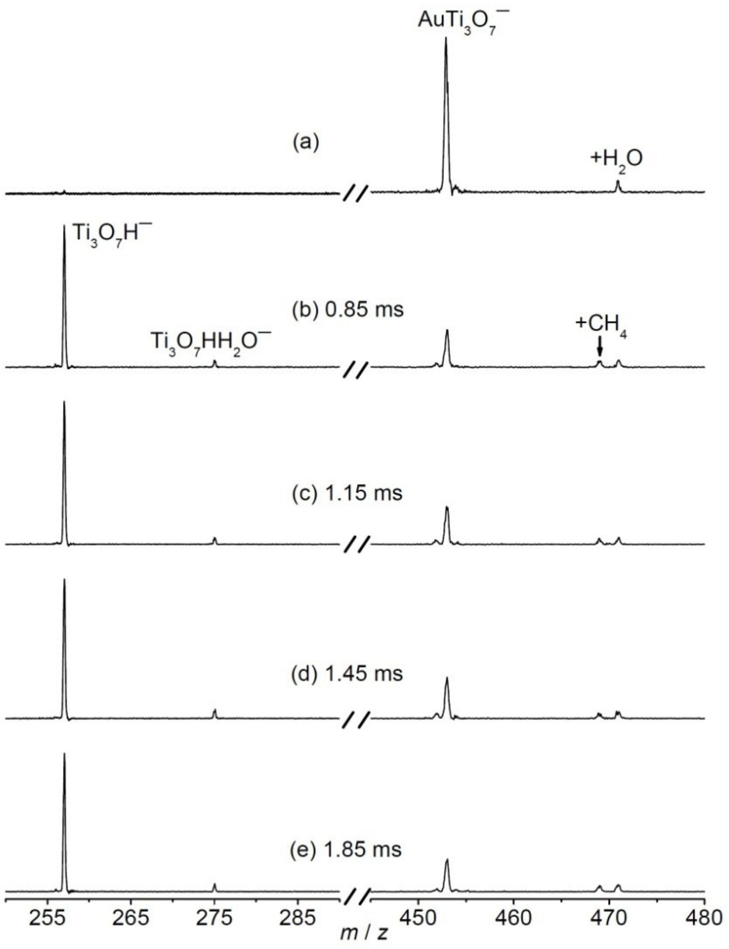

Fig. S4. TOF mass spectra for the reactions of mass selected $AuTi_3O_7^-$ (a) with 95 mPa CH_4 for about 1.07 ms (b–e). The $AuTi_3O_7^-$ cluster ions are cooled for 0.85 ms (b), 1.15 ms (c), 1.45 ms (d), and 1.85 ms (e), respectively.

3. Additional computational results

3.1. Isomeric structures of clusters

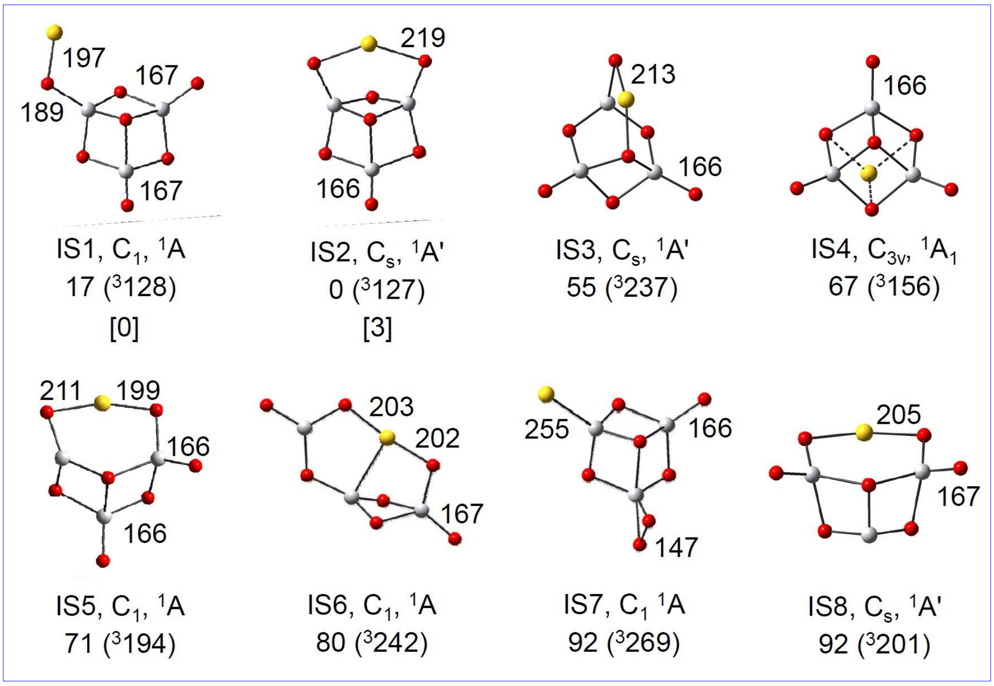

Fig. S5. TPSS optimized structures and relative energies of $AuTi_3O_7^-$ cluster isomers. The energies are in unit of kJ mol^{-1} and bond lengths are in unit of pm. The triplet state energies are given in the *parenthesis*. The CCSD(T) single-point energies at the TPSS optimized isomeric structures are given in the *square brackets* for the two lowest-energy isomers with singlet state.

Theoretical calculations at TPSS level predicted that the most stable structure of $AuTi_3O_7^-$ cluster is the singlet isomer (IS2) with one two-fold coordinated gold cation (Au_{2f}^+) and one terminal-bonded oxygen anions (O_t^{2-}), whereas the singlet isomer (IS1) with one-fold coordinated gold cation (Au_{1f}^+) and two O_t^{2-} ions is higher in energy than IS2 by 17 kJ mol^{-1}. In contrast, the CCSD(T) single-point energy of IS1 calculated at the TPSS optimized structure is lower in energy than that of IS2 by 3 kJ mol^{-1}. Reaction mechanism calculations demonstrated that IS1 is reactive with methane (Fig. 2 in main text), however, IS2 is inert. Based on the experimental observation that the reactive component of the prepared $AuTi_3O_7^-$ clusters accounts for 87% (Fig. S2), we conclude that IS1 is the lowest-lying isomer of the $AuTi_3O_7^-$ cluster.

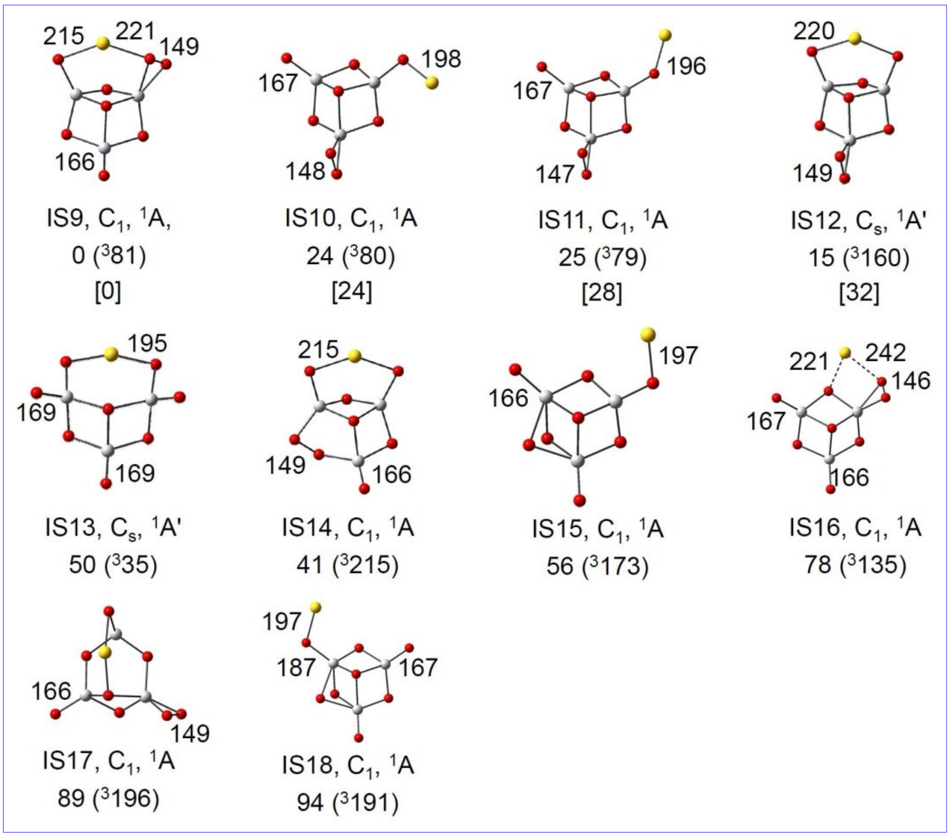

Fig. S6. TPSS optimized structures and relative energies of $AuTi_3O_8^-$ cluster isomers. The energies are in unit of kJ mol^{-1} and bond lengths are in unit of pm. The triplet state energies are given in the *parenthesis*. The CCSD(T) single-point energies at the TPSS optimized isomeric structures are given in the *square brackets* for the four lowest-energy isomers with singlet state.

For $AuTi_3O_8^-$ cluster, the TPSS functional predicted that the most stable structure is IS9 with one Au_{2f}^+ cation and one O_t^{2-} anion. The energies of IS10 and IS11 with one Au_{1f}^+ and one O_t^{2-} are 24−25 kJ mol^{-1} higher than that of IS9. The CCSD(T) single-point energy calculations predicted similar energetics for IS9−IS11. Reaction mechanism calculations demonstrate that both IS10 and IS11 are reactive with methane (Fig. 3 in the main text). However, IS9 and IS12 are inert. Based on the experimental observation that the reactive component of the prepared $AuTi_3O_8^-$ clusters only accounts for 37% (Fig. S3), it can be concluded that IS9 can be the lowest-lying isomer of the $AuTi_3O_8^-$ cluster. The low-lying isomers of IS10 and IS11 correspond to the reactive component of $AuTi_3O_8^-$ in experiments.

3.2. Additional reaction pathways for the reactions of $AuTi_3O_8^-$ + CH_4.

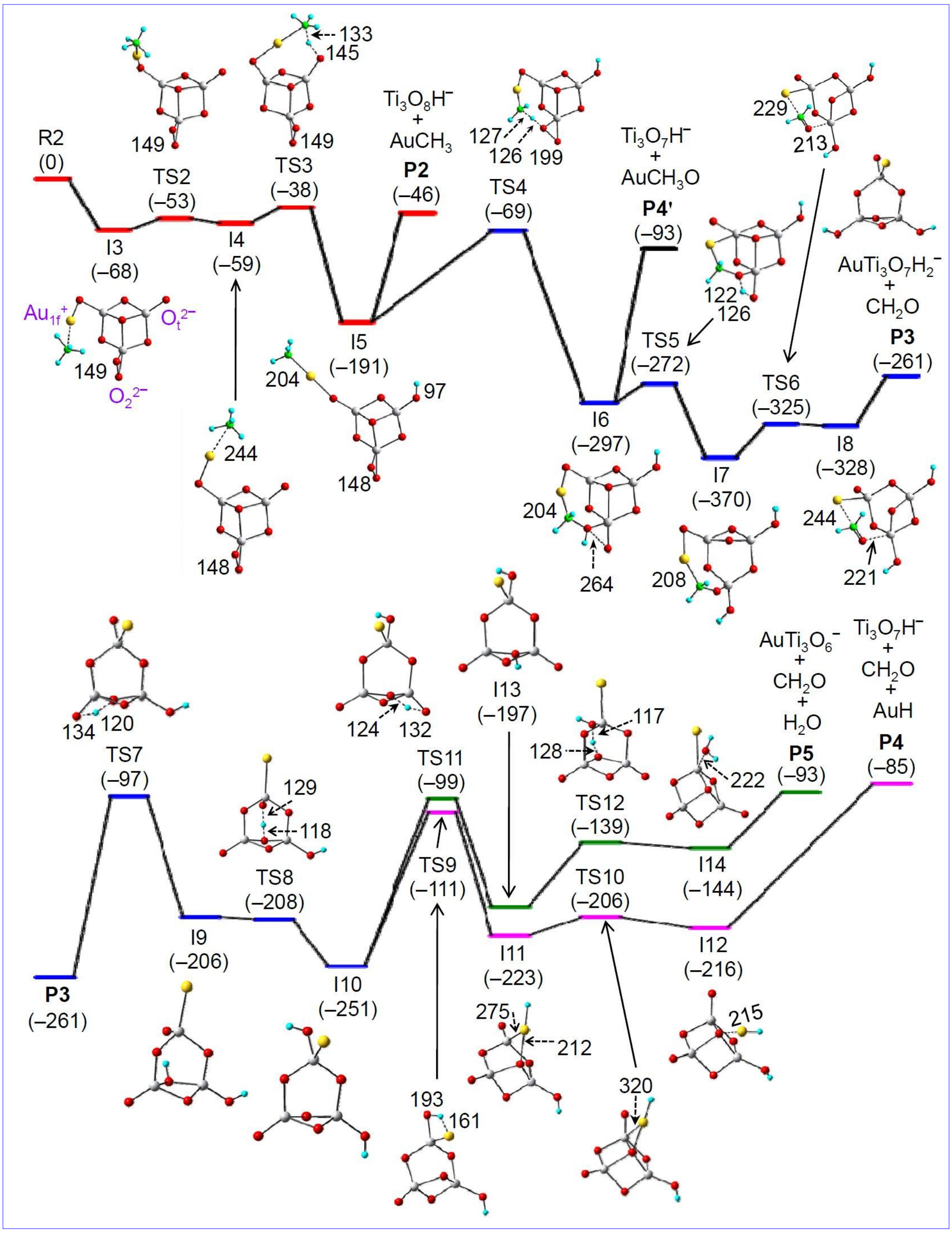

Fig. S7. Complete potential energy surface for $^1AuTi_3O_8^-$ (IS10) + $^1CH_4$ to generate the products of $^1Ti_3O_8H^-$ + $^1AuCH_3$ (P2), $^1AuTi_3O_7H_2^-$ + $^1CH_2O$ (P3), $^1Ti_3O_7^-$ + $^1CH_2O$ + ^1AuH (P4), $^1Ti_3O_7H^-$ + $^1AuCH_3O$ (P4′), and $^1AuTi_3O_6^-$ + $^1CH_2O$ + ^1H_2O (P5). The dispersion corrected energies of the reaction intermediates (I3−I14), transition states (TS2−TS12), and products (P2−P5 and P4′) with respect to the separated reactants [R2, $^1AuTi_3O_8^-$ (IS10) + $^1CH_4$] are given in kJ mol^{-1}. Bond lengths are given in pm. Note that the intermediate I4 can also be formed by adsorption of CH_4 onto $AuTi_3O_8^-$ (IS11).

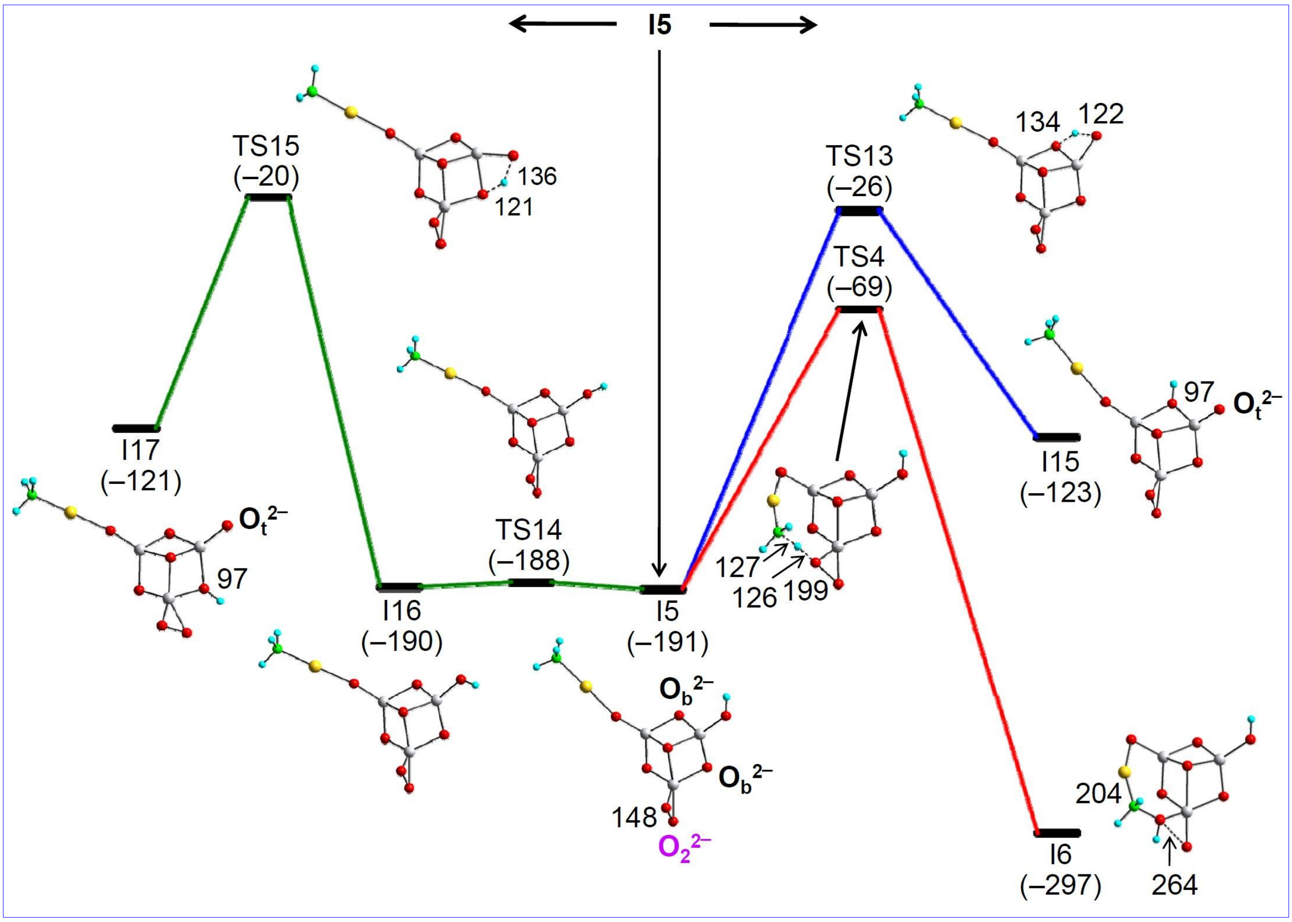

Fig. S8. Possible reaction pathways for complex rearrangement starting from the I5 (Fig. 3 in main text and Fig. S7) in $^1AuTi_3O_8^-$ (IS10) + $^1CH_4$. The dispersion corrected energies of the reaction intermediates (I5, I6, and I15−I17) and transition states (TS4 and TS13−TS15) with respect to the separated reactants [R2, $^1AuTi_3O_8^-$ (IS10) + $^1CH_4$] are given in kJ mol^{-1}. Bond lengths are given in pm.

In the reaction of $AuTi_3O_8^-$ (IS10) + CH_4, the second C−H bond can be activated by the peroxide ion O_2^{2-} (I5 → TS4 → I6). Alternatively, the H atom of OH group in I5 may be transferred to the adjacent bridging O atoms (O_b^{2-}) (I5 → TS13 → I15 and I5 → TS14 → I16) to generate a terminal oxygen atom (O_t^{2-}, see I15 and I17) for activating the second C−H bond. However, the relative energies of TS13 (−26 kJ mol^{-1}) and TS15 (−20 kJ mol^{-1}) are higher than that of TS4 (−69 kJ mol^{-1}). Thus, activation of the second C−H bond by the O_2^{2-} unit is more favourable.

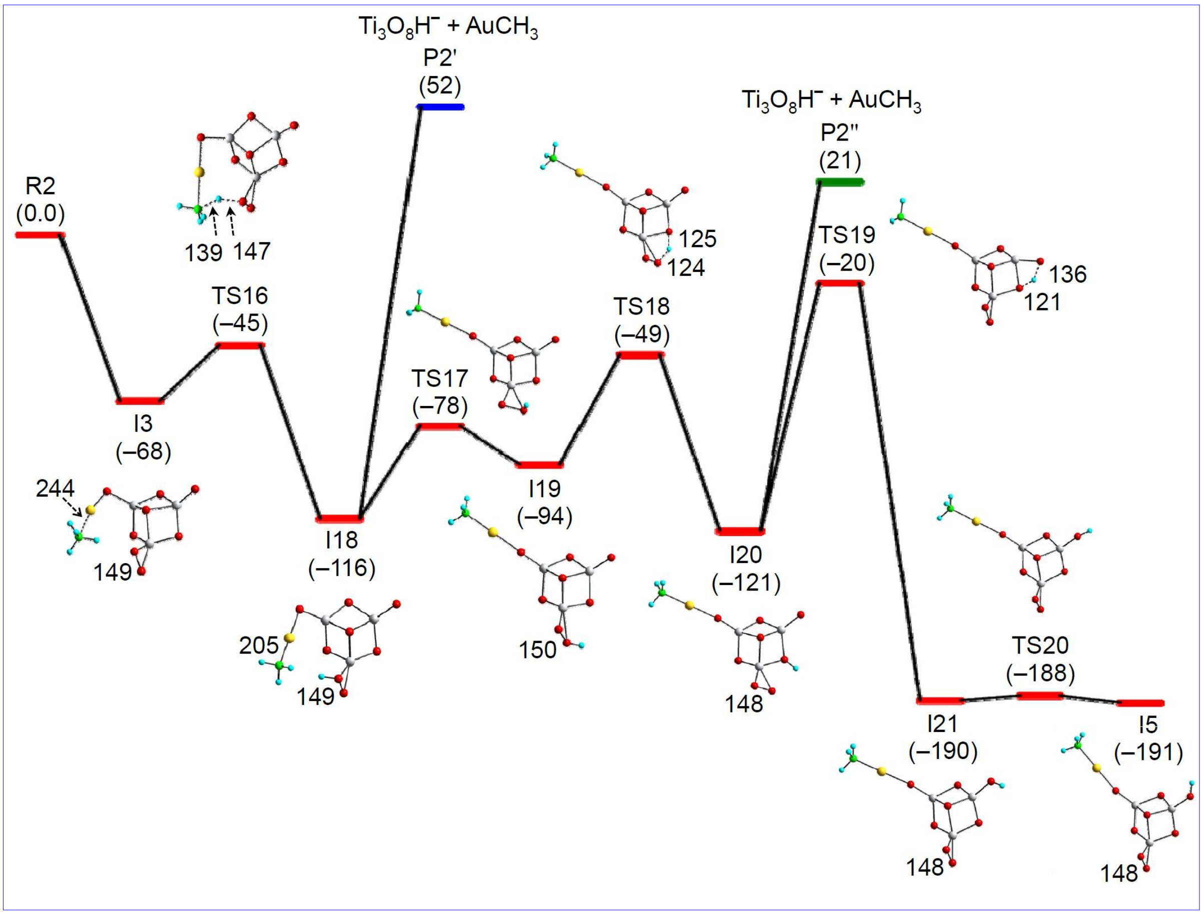

Fig. S9. Additional reaction pathway for formation of intermediate I5 (Fig. 3 in main text and Fig. S7) in $^{1}AuTi_3O_8^-$ (IS10) + $^{1}CH_4$. The dispersion corrected energies of the reaction intermediates (I3, I5, and I18−I21), transition states (TS16−TS20), and products (P2' and P2") with respect to the separated reactants [R2, $^{1}AuTi_3O_8^-$ (IS10) + $^{1}CH_4$] are given in kJ mol^{-1}. Bond lengths are given in pm.

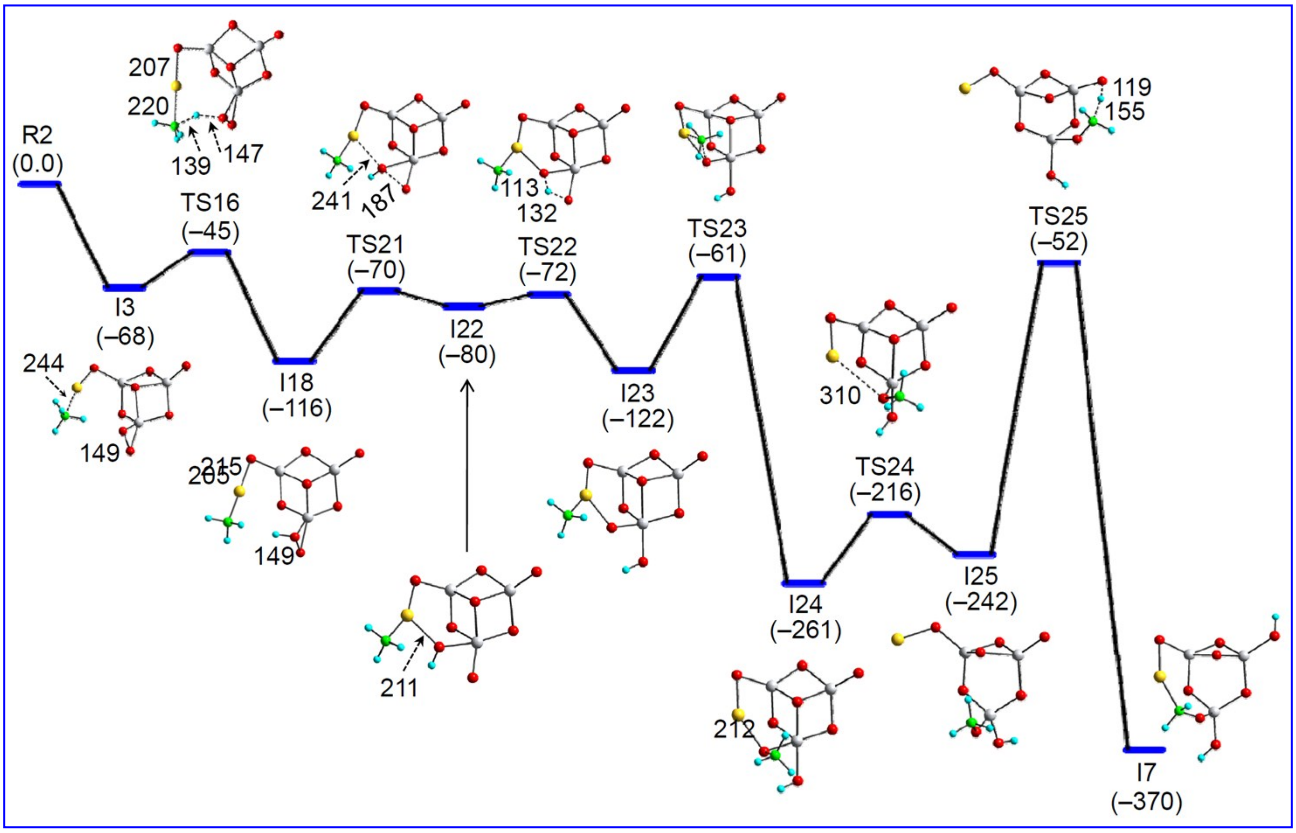

Fig. S10. Additional reaction pathway for formation of intermediate I7 (Fig. 3 in main text and Fig. S7) in $^{1}AuTi_3O_8^-$ (IS10) + $^{1}CH_4$. The dispersion corrected energies of the reaction intermediates (I3, I7, I18, and I22−I25) and transition states (TS16 and TS21−TS25) with respect to the separated reactants [R2, $^{1}AuTi_3O_8^-$ (IS10) + $^{1}CH_4$] are given in kJ mol^{-1}. Bond lengths are given in pm.

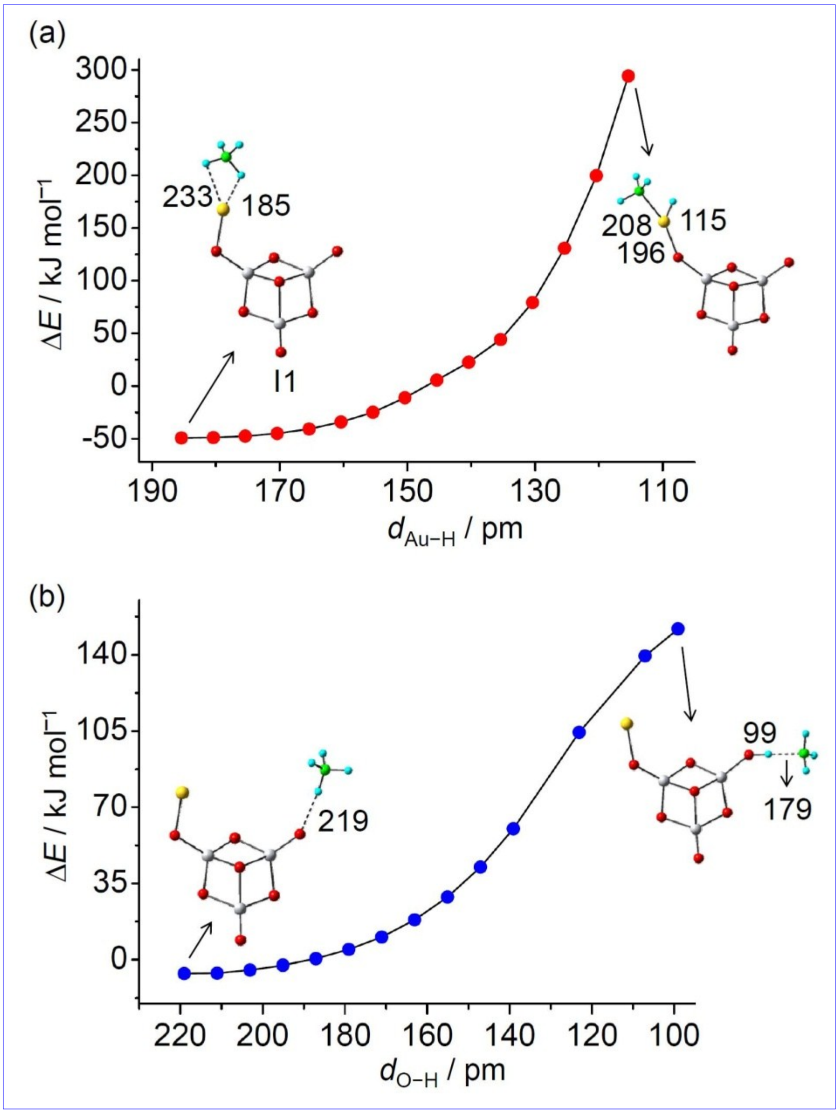

Fig. S11. Reaction pathways for C−H bond activation of methane by single Au_{1f}^{+} ion (a) and single O_t^{2-} ion (b) in $AuTi_3O_7^{-}$ (IS1) along singlet potential energy surface. The energies of the reaction complexes are with respect to the separated reactants [$^{1}AuTi_3O_7^{-}$ (IS1) + $^{1}CH_4$] without zero-point vibrational energy corrections. Bond lengths are given in pm.

3.3. Natural bond orbital analysis of the charge and wiberg bond order of critical atoms and chemical bonds involved in the species of Figure 2 in the main text.

Table S2. NBO charges (e) of Au, O bonded with Au (O_{Au}), transferring H, CH_3 group, and O accepting H (O_H) in the species of Figure 2 at TPSS functional level.

	Au	O_{Au}	CH_3	H	O_H	Ti_3O_7
Reactant	+0.42	−0.75	−0.20	+0.20	−0.62	−1.42
I1	+0.44	−0.81	−0.11	+0.18	−0.63	−1.51
TS1	+0.49	−0.75	−0.31	+0.37	−0.75	−1.54
I2	+0.28	−0.70	−0.41	+0.47	−0.84	−1.34
Product	+0.15	−0.62	−0.15	+0.46	−0.86	−1.46

Table S3. Wiberg bond orders of some important bonds in the species of Figure 2 at TPSS functional level. The Ti atoms bonded with O_{Au} and O_H are denoted as Ti(1) and Ti(2), respectively.

	Au−O_{Au}	Au−C	Ti(1)−O_{Au}	Ti(2)−O_H	C−H	O−H
Reactant	0.83		0.99	2.0	0.96	
I1	0.72		1.03	1.99	0.83	
TS1	0.46	0.34	1.32	1.54	0.53	0.23
I2	0.27	0.78	1.59	0.98		0.76
Product		1.01	2.0	0.95		0.77

3.4 Coordinates for the low-lying energy isomers of $AuTi_3O_7^-$ and $AuTi_3O_8^-$ clusters shown in Figs. S5 and S6. All the coordinates (unit in 0.1 nm) are obtained at TPSS functional level.

$AuTi_3O_7^-$, ^{1}IS1

Au	2.74381000	0.01478900	-0.10831300
Ti	-3.08751600	-0.98154300	-0.12158800
Ti	-0.34757300	-0.62494800	0.38204800
Ti	-2.00479100	1.62544900	0.08141100
O	-2.02099400	3.17884300	-0.53137200
O	-1.51210000	-0.02678700	-1.00976900
O	-1.64587500	-1.74284000	0.95052600
O	-4.16727400	-1.95699500	-0.93726200
O	-3.61531800	0.73166600	0.45364400
O	1.34954800	-1.36963900	0.02368800
O	-0.52344300	0.98757500	1.17999100

$AuTi_3O_7^-$, ^{1}IS2

Au	-0.17459600	-1.83644700	0.00000000
Ti	-0.28378700	2.84429000	0.00000000
Ti	0.42667800	0.48326500	1.37220500
Ti	0.42667800	0.48326500	-1.37220500
O	-0.02686900	-1.12723500	-2.07036100
O	-0.87066100	0.91724500	0.00000000
O	0.42667800	2.30473700	1.74389900
O	-1.44739300	4.02651900	0.00000000
O	0.42667800	2.30473700	-1.74389900
O	-0.02686900	-1.12723500	2.07036100
O	1.67625800	0.35639100	0.00000000

$AuTi_3O_7^-$, ^{1}IS3

Au	-1.28356800	0.52262400	0.00000000
Ti	0.86425200	-1.18775000	1.37257800
Ti	0.86425200	-1.18775000	-1.37257800
Ti	1.00606600	1.72498900	0.00000000
O	1.35443200	0.74455500	-1.45610600
O	-0.60331300	2.54152800	0.00000000

O	1.35443200	0.74455500	1.45610600
O	0.86425200	-2.07010000	2.78388300
O	0.86425200	-2.07010000	-2.78388300
O	1.96465000	-1.82424400	0.00000000
O	-0.64354000	-1.43820000	0.00000000

$AuTi_3O_7^-$, ^{1}IS4

Au	-1.46303600	0.01235700	-0.00005600
Ti	0.89856800	-0.83798200	-1.43264000
Ti	0.89849100	-0.83294600	1.43562400
Ti	0.92555000	1.64874400	-0.00287200
O	1.60846200	3.16444500	-0.00553400
O	2.01832500	-0.02104500	0.00009300
O	0.05373000	-1.86707300	0.00323500
O	1.55642800	-1.60876400	-2.75046300
O	0.08362000	0.93240500	-1.61822200
O	1.55626900	-1.59908800	2.75619800
O	0.08347200	0.93810300	1.61493400

$AuTi_3O_7^-$, ^{1}IS5

Ti	2.47714100	-0.46472700	-0.41658500
Ti	0.64087200	1.75505000	0.19503400
Ti	0.17879100	-1.69699900	0.66777000
O	-1.54941900	-2.03980600	0.38440900
O	1.06524200	3.08183900	1.10272900
O	2.01012400	1.18600800	-1.04042800
O	4.07108800	-0.71444900	-0.00360100
O	1.10360000	-0.13138300	1.09677700
O	1.32355500	-2.11774300	-0.60037100
O	-1.12649800	1.90400100	-0.46024900
Au	-1.61659800	-0.00507400	-0.17279700

$AuTi_3O_7^-$, ^{1}IS6

Ti	-0.26041000	1.63140700	0.00041200
Ti	2.78484700	0.58413600	-0.00079300
Ti	-2.61854700	0.37125100	-0.00005200

O	-1.88975000	-1.31968200	-0.00129100
O	4.44182700	0.77676700	0.00036200
O	1.57011800	2.02462800	-0.00017000
O	-4.28343700	0.47469500	-0.00002900
O	-1.43721900	1.42028500	1.29828100
O	-1.43727500	1.42149500	-1.29749500
O	2.14995100	-1.10937700	0.00146000
Au	0.11590800	-1.09392400	0.00000700

$AuTi_3O_7^-$, ^{1}IS7

Ti	2.38299300	-1.12766000	-0.16460100
Ti	-0.16004300	0.12577800	-0.36862100
Ti	2.06704500	1.73369900	0.05449800
O	2.58671500	3.11461200	0.82126500
O	1.12781200	0.19776400	0.96806800
O	0.75836200	-1.19491700	-1.18294200
O	2.80243500	-2.25556600	1.25683000
O	3.29033500	0.42778000	-0.55787500
O	0.42819000	1.68784000	-1.01032900
O	3.38802500	-2.67760000	-0.04279000
Au	-2.65107400	-0.13290200	0.10777300

$AuTi_3O_7^-$, ^{1}IS8

Ti	-0.37627500	-2.53062000	0.00000000
Ti	-0.50687400	-0.38417600	1.87404200
Ti	-0.50687400	-0.38417600	-1.87404200
O	0.47810600	1.14000100	-2.02893700
O	-1.57498800	-0.57284700	3.13968800
O	0.47810600	-2.13484900	1.47270400
O	-1.57498800	-0.57284700	-3.13968800
O	-1.37687700	-1.03454900	0.00000000
O	0.47810600	-2.13484900	-1.47270400
O	0.47810600	1.14000100	2.02893700
Au	0.65184800	1.34097300	0.00000000

$AuTi_3O_8^−$, [1]IS9

Au	1.81522800	-0.20843200	-0.24276700
Ti	-0.57305400	1.43693300	0.38963900
Ti	-0.51412300	-1.33854100	0.56166800
Ti	-2.87783700	-0.11739100	-0.27177200
O	1.14140400	1.86032100	-0.65420100
O	-4.05756700	-0.24140200	-1.43247900
O	-0.35287500	0.14395600	1.68849300
O	0.84107600	2.70130600	0.53544400
O	-2.38077300	1.66840900	0.37588400
O	1.07151500	-2.17075500	0.22406400
O	-0.96485500	-0.10407000	-0.84170200
O	-2.31951700	-1.74725100	0.63310200

$AuTi_3O_8^−$, [1]IS10

Au	-2.86106300	0.05889700	0.09415800
Ti	2.08654800	1.41395000	-0.22184800
Ti	0.23808000	-0.74436400	-0.35780800
Ti	2.91343000	-1.31522400	0.13546800
O	2.01381100	2.69743800	1.14853800
O	3.92427700	-2.33740300	0.97854200
O	0.58883600	0.82314000	-1.23303100
O	2.39226200	3.25339800	-0.16829500
O	3.53289500	0.33713400	-0.57262600
O	-1.48124700	-1.35957600	0.05426600
O	1.45996200	-0.21742100	0.98170000
O	1.41754300	-2.00281600	-0.89738500

$AuTi_3O_8^−$, [1]IS11

Au	3.08828100	-0.10327600	0.09260200
Ti	-3.03538700	-0.86153900	-0.11759500
Ti	-0.25939700	-0.31432700	-0.36862000
Ti	-2.00921200	1.81873700	-0.00345000
O	-3.73036300	-1.80533400	1.35370300
O	-2.15558700	3.30959900	0.72820800
O	-1.52309000	-1.39898600	-1.12463900

O	-4.40789000	-2.11269900	0.07979200
O	-3.52840300	0.84608800	-0.59458700
O	1.38049000	-1.07603500	0.06508300
O	-1.51411800	0.13569400	0.96992800
O	-0.43182400	1.35363000	-1.04535600

$AuTi_3O_8^-$, [1]IS12

Au	-2.00008900	-0.00000600	-0.27676900
Ti	0.28217300	-1.35932100	0.50818800
Ti	0.28117100	1.35842400	0.50780700
Ti	2.75735400	0.00057100	-0.02555000
O	-1.28040800	-2.06945100	-0.05962100
O	3.57741900	-0.00095100	-1.68968100
O	0.01336000	-0.00037800	1.74364400
O	4.53839400	0.00134700	-0.55370000
O	2.13025100	-1.65703100	0.68373400
O	-1.28135200	2.06980700	-0.05815900
O	0.79247200	-0.00060200	-0.73951700
O	2.12882100	1.65821700	0.68267100

$AuTi_3O_8^-$, [1]IS13

Ti	-2.05540500	1.50171900	0.00000000
Ti	-0.01882900	0.59974100	1.79947000
Ti	-0.01882900	0.59974100	-1.79947000
O	0.66630000	-1.20504300	-1.91837100
O	0.66630000	1.51595500	3.04731200
O	-1.88452700	0.67480100	1.69583400
O	-2.82385800	3.00741900	0.00000000
O	-0.05518700	1.47275300	0.00000000
O	-1.88452700	0.67480100	-1.69583400
O	0.66630000	-1.20504300	1.91837100
Au	0.98621000	-1.40555900	0.00000000
O	0.66630000	1.51595500	-3.04731200

$AuTi_3O_8^-$, [1]IS14

Au	-1.94544200	-0.02318200	-0.22070800

Ti	0.30100900	-1.33428000	0.56295600
Ti	0.46054400	1.42873600	0.28378600
Ti	2.80806000	-0.42710200	-0.27301300
O	0.93204100	-0.09167300	-0.84332700
O	3.91469400	-0.70380600	-1.47705500
O	0.25358600	0.14360600	1.66427200
O	-1.30951200	-2.04551100	0.14124200
O	2.04962600	-1.96611900	0.57960400
O	-1.16355500	1.98407200	-0.19926800
O	2.00859900	2.49215100	0.05810100
O	2.70932400	1.33097600	0.67817100

$AuTi_3O_8^-$, [1]IS15

Au	-2.90057100	0.02906500	-0.09202000
Ti	1.91431500	1.58441500	0.01110400
Ti	0.20747000	-0.63682300	0.34634700
Ti	2.94692900	-1.07512500	-0.15381000
O	3.46267800	0.59164500	0.99385500
O	1.33461800	-0.10255800	-1.06446100
O	3.73248400	0.78865600	-0.47340500
O	1.80811800	3.18365700	-0.41745200
O	3.94276800	-2.25544300	-0.75564200
O	-1.51189900	-1.35955500	0.02536400
O	1.49490000	-1.76607900	0.92429600
O	0.44050500	0.98337900	1.11612400

$AuTi_3O_8^-$, [1]IS16

Au	2.33982300	0.17410500	-0.08686900
Ti	-0.47422700	-1.17127700	0.06650300
Ti	-1.19205200	1.63359500	0.09599000
Ti	-3.16731800	-0.37009700	-0.21183800
O	1.34608800	-2.03501700	-0.02771600
O	-1.65223400	-0.04919400	1.10873100
O	-1.89001600	-1.70570300	-0.88513100
O	0.54177400	-2.37300000	1.14150000
O	-4.70411000	-0.79657100	0.26621400

O	-0.84938300	3.09684300	0.82164800
O	0.22332300	0.50132000	-0.64170900
O	-2.82879900	1.38843100	-0.79000800

$AuTi_3O_8^-$, [1]IS17

Au	-1.23061400	-0.43231700	-0.68101700
Ti	1.89051200	-0.66638400	0.22551800
Ti	0.73190600	1.85708400	-0.19272800
Ti	-0.90061500	0.02572500	1.82186100
O	-0.50064200	1.70692200	1.36677400
O	-2.40345600	-0.64730300	1.10604100
O	2.60947700	-1.89734400	-1.00431400
O	0.58547900	-0.98362700	1.64377800
O	3.30419400	-1.85330000	0.30973600
O	0.84437900	3.32155000	-0.97359700
O	2.34337900	1.09497400	0.45292400
O	0.63455100	0.18208600	-1.27659000

$AuTi_3O_8^-$, [1]IS18

Au	2.78336000	0.00357000	-0.10994900
Ti	-0.31625400	-0.65953000	0.29005300
Ti	-1.73671600	1.75501500	0.13853700
Ti	-3.11857200	-0.73519500	-0.23210100
O	-1.83976900	-1.44612000	1.32312900
O	-1.38651200	0.16681800	-1.04333900
O	-1.62881500	-2.19906500	0.03120600
O	1.37819400	-1.38158300	-0.02216900
O	-4.45345000	-1.41334600	-0.94869200
O	-1.58749000	3.33169900	-0.39980900
O	-3.42977300	0.99607300	0.42958400
O	-0.31632300	0.91946900	1.17549100

References

1 Z. Yuan, Z.-Y. Li, Z.-X. Zhou, Q.-Y. Liu, Y.-X. Zhao and S.-G. He, *J. Phys. Chem. C*, 2014, **118**, 14967−14976.

2 M. J. Frisch, G. W. Trucks, H. B. Schlegel, G. E. Scuseria, M. A. Robb, J. R. Cheeseman, G. Scalmani, V. Barone, B. Mennucci, G. A. Petersson, H. Nakatsuji, M. Caricato, X. Li, H. P. Hratchian, A. F. Izmaylov, J. Bloino, G. Zheng, J. L. Sonnenberg, M. Hada, M. Ehara, K. Toyota, R. Fukuda, J. Hasegawa, M. Ishida, T. Nakajima, Y. Honda, O. Kitao, H. Nakai, T. Vreven, J. A. Montgomery, Jr., J. E. Peralta, F. Ogliaro, M. Bearpark, J. J. Heyd, E. Brothers, K. N. Kudin, V. N. Staroverov, R. Kobayashi, J. Normand, K. Raghavachari, A. Rendell, J. C. Burant, S. S. Iyengar, J. Tomasi, M. Cossi, N. Rega, J. M. Millam, M. Klene, J. E. Knox, J. B. Cross, V. Bakken, C. Adamo, J. Jaramillo, R. Gomperts, R. E. Stratmann, O. Yazyev, A. J. Austin, R. Cammi, C. Pomelli, J. W. Ochterski, R. L. Martin, K. Morokuma, K.,V. G. akrzewski, G. A. Voth, P. Salvador, J. J. Dannenberg, S. Dapprich, A. D. Daniels, O. Farkas, J. B. Foresman, J. V. Ortiz, J. Cioslowski and D. J. Fox, Gaussian 09 (Revision A.01), Gaussian, Inc., Wallingford CT, 2009.

3 A. Schäfer, C. Huber and R. Ahlrichs, *J. Chem. Phys.* 1994, **100**, 5829−5835.

4 M. Dolg, H. Stoll and H. Preuss, *J. Chem. Phys.* 1989, **90**, 1730−1734.

5 J. Tao, J. P. Perdew, V. N. Staroverov and G. E. Scuseria, *Phys. Rev. Lett.* 2003, **91**, 146401.

6 A. D. Becke, *Phys. Rev. A* 1988, **38**, 3098−3100.

7 C. Lee, W. Yang and R. G. Parr, *Phys. Rev. B* 1988, **37**, 785−789.

8 A. D. Becke, *J. Chem. Phys.* 1993, **98**, 5648−5652.

9 X.-N. Li, Z. Yuan and S.-G. He, *J. Am. Chem. Soc.* 2014, **136**, 3617−3623.

10 J.-B. Ma, B. Xu, J.-H. Meng, X.-N. Wu, X.-L. Ding, X.-N. Li and S.-G. He, *J. Am. Chem. Soc.* 2013, **135**, 2991−2998.

11 G. D. Purvis III and R. J. Bartlett, *J. Chem. Phys.* 1982, **76**, 1910−1918.

12 G. E. Scuseria, C. L. Janssen and H. F. Schaefer III, *J. Chem. Phys.* 1988, **89**, 7382−7387.

13 J. A. Pople, M. Head-Gordon and K. Raghavachari, *J. Chem. Phys.* 1987, **87**, 5968−5975.

14 H. B. Schlegel, *J. Comput. Chem.* 1982, **3**, 214−218.

15 E. D. Glendening, A. E. Reed, J. E. Carpenter and F. Weinhold, NBO, version 3.1, 1996.

16 J. B. Pedley and E. M. Marshall, *J. Phys. Chem. Ref. Data* 1983, **12**, 967−1031.

17 K. P. Huber and G. Herzberg, Molecular Spectra and Molecular Structure. IV. Constants of Diatomic Molecules Van Nostrand Reinhold: New York, 1979.

18 F. Aguirre, J. Husband, C. J. Thompson and R. B. Metz, *Chem. Phys. Lett.* 2000, **318**, 466–470.

19 G. L. Gutsev, L. Andrews and C. W. Bauschlicher, Jr., *Theor. Chem. Acc.* 2003, **109**, 298−308.

20 http://webbook.nist.gov/chemistry.

21 K. M. Ervin, I. Anusiewicz, P. Skurski, J. Simons and W. C. Lineberger, *J. Phys. Chem. A* 2003, **107**, 8521−8529.

22 D. R. Lide, Handbook of Chemistry and Physics; CRC Press LLC, 2003, p9−53.
